# “*She was totally desperate”*: understanding the pathway to abortion in Germany through a qualitative study among service providers in Berlin and Brandenburg

**DOI:** 10.1080/26410397.2025.2534266

**Published:** 2025-07-23

**Authors:** Talia Meer, Mascha Kern, Alex Müller, Bornice Biomndo, Yagmur Demirpehlivan, Muhammad Helmi Barghouth, Stefanie Theuring

**Affiliations:** a Research Associate, Department of History, Humboldt Universität zu Berlin, Berlin, Germany; Adjunct senior researcher, Gender Health and Justice Research Unit, University of Cape Town, Cape Town, South Africa; b Research Associate, Institute of Public Health, Charité – Universitätsmedizin Berlin, corporate member of Freie Universität Berlin and Humboldt Universität zu Berlin, Berlin, Germany; c Research Associate, Institute of International Health, Center for Global Health, Charité – Universitätsmedizin Berlin, corporate member of Freie Universität Berlin and Humboldt Universität zu Berlin, Berlin, Germany; Adjunct Associate Professor, Gender Health and Justice Research Unit, University of Cape Town, Cape Town, South Africa; d Research Associate, Institute of International Health, Center for Global Health, Charité – Universitätsmedizin Berlin, corporate member of Freie Universität Berlin and Humboldt Universität zu Berlin, Berlin, Germany; e Senior Research Associate, Institute of International Health, Center for Global Health, Charité – Universitätsmedizin Berlin, corporate member of Freie Universität Berlin and Humboldt Universität zu Berlin, Berlin, Germany.

**Keywords:** abortion, reproductive rights, Germany, German Penal Code §218a, providers, qualitative research

## Abstract

Access to abortion is recognised as a key component of the right to healthcare. Yet, to this day, abortion in Germany without medical or criminal indication is illegal and treated under the penal code, although not punished if taking place in the first 12 gestational weeks and following a formalised process including mandatory counselling. Our study aimed to critically examine the pathway to abortion necessitated by the current criminalising legal framework in Germany. We conducted a qualitative study between November 2023 and July 2024, using 17 interviews to capture views of abortion-related service providers on barriers and enablers to abortion in Berlin and Brandenburg, applying a thematic analysis approach using MaxQDA. Our findings point to multiple individual barriers to abortion along the pathway, whose complexity itself presents a structural barrier. Even providers were sometimes insecure about how parts of the process worked and what they were or were not allowed to do. We identified a nine-step series of actions which most individuals seeking abortion must undergo to end an unwanted pregnancy, involving multiple health service visits and the repeated need to communicate their personal situation to different authorities. Our study illustrates that the pathway to abortion in Germany produces a state-induced barrier to a fundamental reproductive health service, and in this respect can be viewed as a form of structural violence against pregnant individuals in a critically decisive situation. Our findings support existing recommendations to decriminalise abortion, which would align Germany´s abortion policy with its international commitments and with international best practices as outlined by WHO.

## Plain language summary

Access to abortion is an important part of healthcare rights. In Germany, abortion is illegal unless it is for medical or criminal reasons. If done in the first 12 weeks of pregnancy and following a specific process, it will not be punished. But this process is complicated and involves mandatory counselling. Our study aimed to explore how this legal framework affects the process of getting an abortion in Germany. We interviewed 17 abortion-related service providers in Berlin and Brandenburg to understand the challenges and supports in the abortion process. The interviews were analysed for recurring themes using MaxQDA software. We found that the abortion care process is complicated, even the providers were sometimes unclear about what it involved or what was permitted. Pregnant individuals typically must go through a nine-step procedure before they can get an abortion. This includes multiple health visits and explaining their situation to various authorities. Our research shows that the current legal framework creates unnecessary obstacles to a basic health service. This can be seen as a form of structural violence against pregnant individuals who are facing a difficult situation with major implications for their future lives. Our findings support calls to decriminalise abortion in Germany. This would align its policies with international standards and best practices set by the World Health Organization.

## Résumé

L’accès à l’avortement est reconnu comme un élément clé du droit à la santé. Pourtant, à ce jour, l’avortement en Allemagne sans indication médicale ou pénale est illégal et relève du code pénal. Il n’est cependant pas sanctionné s’il est pratiqué au cours des 12 premières semaines de grossesse et s’il fait suite à une procédure formalisée comprenant une consultation obligatoire. Notre étude visait à examiner de manière critique le parcours vers l’avortement requis par le cadre juridique pénalisant actuellement en vigueur en Allemagne. Nous avons mené une étude qualitative à partir de 17 entretiens entre novembre 2023 et juillet 2024, afin de recueillir le point de vue des prestataires de services liés à l’avortement sur les obstacles et les facteurs facilitant l’interruption de grossesse à Berlin et dans le Brandebourg, en appliquant une approche d’analyse thématique qui utilise MaxQDA. Nos résultats mettent en évidence de multiples obstacles individuels à l’avortement tout au long du parcours, dont la complexité constitue elle-même une barrière structurelle. Même les prestataires de services étaient parfois incertains quant au fonctionnement du processus et à ce qu’ils étaient ou non autorisés à faire. Nous avons identité une série de neuf étapes que la plupart des personnes souhaitant avorter doivent suivre pour interrompre une grossesse non désirée, avec de multiples visites à des services de santé et la nécessité répétée de communiquer leur situation personnelle à différentes autorités. Notre étude montre que le parcours vers l’avortement en Allemagne crée un obstacle, induit par l’État, à l’accès à un service fondamental de santé reproductive et peut, à cet égard, être considéré comme une forme de violence structurelle à l’encontre des personnes enceintes dans une situation critique. Nos conclusions étayent les recommandations existantes prônant la dépénalisation de l’avortement, ce qui alignerait la politique allemande en la matière sur ses engagements internationaux et sur les meilleures pratiques internationales définies par l’OMS.

## Resumen

El acceso al aborto es reconocido como un componente clave del derecho a servicios de salud. Sin embargo, hasta la fecha, en Alemania el aborto sin indicación médica o penal es ilegal y tratado bajo el código penal, aunque no es punible si se realiza en las primeras 12 semanas de gestación y después de un proceso formalizado que incluye asesoramiento obligatorio. Nuestro estudio buscaba examinar críticamente la vía al aborto requerida por el actual marco jurídico penalizador de Alemania. Realizamos un estudio cualitativo entre noviembre de 2023 y julio de 2024 utilizando 17 entrevistas para capturar los puntos de vista de los prestadores de servicios de aborto sobre los obstáculos y facilitadores de aborto en Berlín y Brandemburgo, aplicando el enfoque de análisis temática con MaxQDA. Nuestros hallazgos señalan múltiples obstáculos individuales al aborto a lo largo de la vía, cuya complejidad en sí presenta una barrera estructural. Incluso los prestadores de servicios a veces no estaban seguros de cómo funcionan ciertas partes del proceso y qué se les permite o no hacer. Identificamos una serie de acciones de 9 pasos que la mayoría de las personas que buscan un aborto deben realizar para interrumpir un embarazo no deseado, que implican múltiples visitas a los servicios de salud y la necesidad repetida de comunicar su situación personal a diferentes autoridades. Nuestro estudio muestra que la vía al aborto en Alemania produce un obstáculo inducido por el Estado a un servicio de salud reproductiva fundamental, y en este sentido puede considerarse como una forma de violencia estructural contra las personas embarazadas en una situación críticamente decisiva. Nuestros hallazgos apoyan las recomendaciones actuales de despenalizar el aborto, lo cual alinearía la política de Alemania relativa al aborto con sus compromisos internacionales y con las prácticas óptimas internacionales, descritas por la OMS.

## Background

Access to abortion is recognised as a key component of the right to access to healthcare and is anchored in numerous international human rights treaties, as well as within the World Health Organization (WHO)’s guidelines.^1–3^ Nevertheless, the legal framework governing abortion access in Germany does not conform to the standards laid out in these treaties and in international guidelines, and abortion remains criminalised.^4^ So far, Germany regulates abortion under the German Penal Code, § 218ff, according to which abortion is generally illegal but is not punishable if performed within the first 12 weeks after conception and after the abortion-seeker has undergone mandatory counselling.^5^ Exceptions to the 12-week rule include medical necessity or criminal indications, such as sexual assault.^6^ In 2023, 96% of all abortions in Germany were conducted after mandatory counselling and 4% were conducted because of medical necessity or a criminal indication.^7^ The procedure of accessing abortion services is set out in the *Schwangerschaftskonfliktgesetz* (SchKG), the law governing pregnancy conflict.^6^ Firstly, individuals must attend the mandatory counselling at either a state-recognised centre or with a registered gynaecologist.* According to the SchKG § 5,^1^ the counselling session should not have a predetermined outcome; however, the stated overarching aim of the mandatory counselling is to “protect the unborn life”.^6^ The counselling session includes medical, social, and legal information, as well as information about support services available to the pregnant person should they decide to continue the pregnancy. After receiving a counselling certificate, individuals need to wait for three days before the actual abortion procedure can take place; the provider must be completely distinct from the one providing the mandatory counselling. Because abortions under the mandatory counselling rule are criminalised, they are not covered by health insurance and are subject to self-payment,^8^ except in cases of financial hardship, where the federal states cover costs.

Public debates surrounding the decriminalisation of abortion in Germany, which have been ongoing since the 1970s, were sparked again in 2022 with the government overriding § 219a, which criminalised doctors who publicly stated which abortion methods they offered.^6^ In the course of the abolishment of § 219a, the decades-old debate about general decriminalisation of abortion in Germany was revived and led the government to constitute the Commission on Reproductive Self-Determination in March 2023.^9^ In their report, published in April 2024, the Commission declared that the criminalisation of abortion, especially in the early stages of pregnancy, was untenable. In particular, the fact that, according to the current regulations, individuals seeking abortion in Germany are acting unlawfully was viewed as ethically unacceptable.^10^ The Commission recommended that abortion, at least up to 12 weeks, should be regulated outside of the Penal Code, and that abortion access should be more equitable and less bureaucratic.

There is little data on the experiences of individuals who seek abortions in Germany, although there are a few notable studies from recent years. For example, the ELSA study, funded by the German Ministry of Health in 2023, collected data on the provision of care for unintended pregnancies among 5,000 pregnant women. It found that 60% of women seeking abortion care faced organisational barriers, and that one in four had to contact more than one facility to find a doctor to perform the abortion.^11^ In line with these findings, a recent discussion paper concludes that the criminalisation of abortion creates multiple obstacles both for people seeking abortions as well as doctors offering abortions.^12^ Another study examining the use of telemedicine for abortion care (i.e. enabling a medical abortion at home, while being advised throughout the process by video or telephone), showed that telemedicine was increasingly popular among people seeking abortions in Germany, with 60% of persons contacting the services choosing telemedicine because of a lack of abortion facilities where they lived.^13,14^ Another study revealed that the motivations of those turning to telemedicine (outside of the formal German healthcare system) included a desire for autonomy, the need for secrecy, avoiding stigma, financial stress, logistical barriers to access, or provider attitudes.^15^

This points to a serious lack of appropriate in-person services for the specific needs of abortion-seekers. Moreover, this lack of services regarding an internationally recognised human health right could also indicate a form of structural violence, which is present when state policies or legislation create hardships and injustice among vulnerable groups without ameliorating their negative consequences.^16,17^ Structural violence is institutionalised and functions through the everyday operations of healthcare, creating systematic and indirect adversity, and other researchers have recognised it in inadequate abortion care before.^18^

Overall, there is still a knowledge gap concerning barriers to abortion care, particularly those faced by more vulnerable and marginalised population groups in Germany.^19^ Also, abortion research typically locates injustice at the individual level, as a determinant or outcome of seeking care, while the massive structural barrier inherent to the German regulatory framework is often neglected.^20^

This study was conducted in the larger frame of a research project initially focussing on migrant-specific access barriers to healthcare. When examining specific abortion care barriers for recent migrants, it became apparent that numerous structural barriers in fact applied to all abortion seekers. Hence, the aim of this sub-study was to describe the general pathway to abortion necessitated by the current criminalising legal framework in Germany from the perspectives of providers and client advocates across the course of abortion care.

## Methods

We conducted a qualitative study using in-depth interviews to capture service providers' views on barriers and enablers to abortion care in Germany, focusing on the federal states of Berlin and Brandenburg. Berlin has almost 4 million inhabitants and is the most populous city in the European Union, while Brandenburg is a large rural state that has only 2.5 million inhabitants.^21^ By studying these two states, we could draw conclusions about both urban and rural areas in Germany, particularly within the Berlin and Brandenburg region.

Data collection took place between November 2023 and July 2024. We conducted semi-structured in-depth interviews with individuals providing abortion-related services before, during and after termination of pregnancy. As we sought to understand structural barriers, we determined that service providers would provide the best overview of the healthcare system and its pathways and blockages, as they mediate between bureaucratic processes and client needs. Only a few studies have researched the perspective of abortion counsellors, who are vital service providers and gatekeepers to abortion care in Germany.^22^ To identify interview participants, we mapped services of the two states to identify all state-registered organisations offering mandatory counselling.^23^ Based on this, we interviewed those doing mandatory counselling, representing a pivotal and under-researched group for abortion care in Germany. Participants in these initial interviews, selected as a convenience sample in a maximum variability approach to purposely cover many different perspectives, referred us to more interviewees. In addition to counsellors, we sought interviews with doctors performing abortions and abortion advocates. Interviews were conducted depending on participant preference, via video call or in-person. In-person interviews took place in the interviewee’s workplace. Researchers conducting the interviews had no ties to the interviewees and reflected on personal experiences and characteristics that might influence the research before the start of the data collection. All interviews, except one, were conducted by a team of two researchers. All interviewers identify as women and have experience working in sexual and reproductive health and rights. Two are racialised recent migrants to Germany, one is a white woman who migrated to Germany, and two are white German-born women.

An interview guide was developed based on preliminary literature research on relevant aspects, using mostly open-ended questions (Supplementary File S1). Interviews began with collecting information on the organisation where the interviewee worked, their role within it, and what the organisation offered. We then explored their experiences in providing abortion-related care, asking about barriers for recent migrants, but also about barriers applicable to the general population in Germany. Experiences that did not pertain to all abortion seekers, but only to recent migrants, were extracted for a separate analysis, not shown in this sub-study.

According to the interviewees’ preferences, interviews were conducted in either German or English, recorded, and transcribed verbatim using the software Turboscribe (https://turboscribe.ai). Before analysis, transcripts in German were translated into English using DeepL (https://www.deepl.com/de/translator) and proofread for consistency. The research team comprised native English speakers as well as native German speakers with high English proficiency. This enabled us to conduct, proofread and edit interviews and translations in both languages.

We applied a thematic analysis approach.^24^ Transcripts were read for common themes, with a focus on identifying individual, systemic, and structural barriers and gaps in access.^25^ Themes were developed and applied to data using MaxQDA.^26^ The analysis was conducted collaboratively to ensure rigour and consistency. Initially, one author performed the primary coding, which was then reviewed and cross-checked by a second author to assess intercoder reliability. Additionally, all authors discussed the final framework, refining interpretations and aligning on emerging themes.

Throughout this text, the terms “counsellor” and “counselling” refer to mandatory counselling services, according to the German regulatory framework. We continued with interviews until we reached data saturation on barriers and enablers to abortion care. Interviews, transcription, translation and thematic analysis were conducted concurrently to continuously assess the saturation of the results. Thematic saturation was determined when no new barriers or enablers emerged from additional interviews, following a thematic coding process.

### Ethics approval and consent to participate

All participants provided written informed consent prior to the interview. Confidentiality and anonymity were ensured, and interviewees could stop participation at any time. They were also asked to review their transcripts and retract any information they were no longer comfortable sharing. The study was approved by the ethics committee of Charité – Universitätsmedizin Berlin, Vote EA4/152/23, on August 30th, 2023.

## Results

Data in this paper are drawn from 17 in-depth interviews: nine providers of mandatory counselling (seven social workers, two gynaecologists; their workplaces included one Berlin district health department and eight non-profit organisations, partly funded through the state of Berlin); one doctor performing abortions at a practice; five civil society advocates for abortion access (including from pro-choice, sex worker, migrant, drug use organisations, and a support group for people who have experienced abortion); and two individuals working in healthcare translation services used widely in abortion service provision. The sample we interviewed included both native German speakers and individuals who had migrated to Germany. The interviews lasted between 47 and 90 minutes (mean duration: 58 minutes). For details of interviewees, see Table 1.

**Table 1.** Sociodemographic and professional information on interview partners

Inter-view no.	Institution type	Location	Age decade^1^	Gender	Professional role	Highest completed level of Education	Work Experience (years)	Migrant Back-ground
01	Counselling Centre	Berlin	60	Female	MD/Counsellor	University	19	No
02	Centre for Sexual Health and Family Planning	Berlin	60	Female	Social Worker	University	41	No
03	Public Health Department	Berlin	50	Female	MD/Counsellor	University	33	No
04	Public Health Department	Berlin	50	Female	Social Worker/ Counsellor	University	16	No
05	Family Planning Centre	Berlin		Female	MD/Counsellor	University		
06	Counselling Centre	Brandenburg	30	Female	Social Worker/ Counsellor	University	11	No
07	Counselling Centre	Berlin	30	Female	Social Worker/ Client Advocate	University	8	Yes
08	Counselling Centre	Brandenburg	50	Female	Counsellor	University	24	No
09	Counselling Centre	Brandenburg	50	Female	Counsellor	University	25	
10	Counselling Centre	Brandenburg	20	Female	Counsellor	University	3,5	No
11	Self Support Group	Berlin		Female	Client Advocate		7	Yes
12	Activist group	Berlin	20	Female	Medical Student/Client Advocate	High School	3	No
13	NGO	Berlin	30	Female	Counsellor	University		No
14	Counselling Centre		40	Female	Medical Assistant	Middle School	25	Yes
15	Counseling Centre	Berlin	50	Female	Counsellor	University	2	Yes
16	Language Mediation	Berlin	30	Female	Client Advocate/ Translation coordinator	University	8	
17	Language Mediation	Berlin	40	Female	Paramedic/ Translator	University	5	Yes

^1^Age of interview partner is presented within the respective decade, to warrant anonymity of the interview partners.

Based on interview data, seeking an abortion in Germany within the legal framework set out above typically entails a pathway of up to nine steps, partly stipulated by §§ 218 and 219 of the Criminal Code (steps 2, 3, 5):

1. Access information: Learn about abortion laws, rights, and procedures in Germany
2. Determine pregnancy week: Visit a doctor to confirm the pregnancy and determine the gestational age
3. Mandatory counselling: Attend counselling and receive a certificate
4. Find a doctor for an abortion procedure: Locate a doctor who performs abortions and make an appointment
5. 3-day waiting period: Wait for three days after the counselling session before the abortion can be performed
6. Financial arrangements: Arrange for payment by state via health insurance, if income is below the threshold of self-payment
7. Logistic arrangements: Make necessary private and logistic arrangements, as necessary
8. Pre-surgery medical consultation: only in case of surgical abortion
9. Abortion: Undergo the abortion procedure

Several of the interviewees emphasised that the process itself was a barrier to accessing abortion, and that constantly having to meet new people and revealing the need for an abortion was stressful and required personal courage. Our interviews showed that throughout the nine steps, individuals must interface with numerous administrators and service providers, who each have the capacity to facilitate or hinder access to care, also signifying a dependence on personal effort or capacity, as well as moral beliefs or judgements of individual providers:

*“It always depends on who's sitting there. That's always the problem. And it really shouldn't be that hard […].”* (Interviewee 07, social worker and client advocate, Berlin)

Results from our interviews are presented along the line of these consecutive steps of the abortion pathway to demonstrate how they individually and cumulatively act as barriers to abortion access.

### Accessing information

Our interviewees stated that above all, the complex pathway requires that abortion-seekers must first gain reliable information about the legally required steps. This can be challenging, as most providers refer precisely to the legal clauses that provide for access to abortion rather than using accessible plain language, and information is often not apparent on the service providers’ homepages but requires searching through the website. Even doctors who perform abortions usually do not provide this information on their websites, as interviewees point out:

*“[People find information] through contacts, friends, relatives, other people who know about it. Or you can find it online if you search for it. It's often a big hurdle and often difficult to understand all these legal regulations and to understand which paper I need, why and where can I get it? […] the German system, it's not that uncomplicated.”* (Interviewee 05, counsellor, Berlin)

*“As far as communication is concerned, when I look at the websites of the [name] day clinic, for example, which is a very large centre, they do fertility, gynaecology, but also abortions, and abortions, for example, are not communicated there at all. This is a very good website, you can read a lot [of information], but it's difficult to find out about abortion.”* (Interviewee 10, counsellor, Brandenburg)

*“How would the person know that the health insurance or health insurance company will cover the costs? How would they know that? How would you find information on where to do it, because there's no doctor with that on the homepage.”* (Interviewee 07, social worker and client advocate, Berlin)

### Mandatory counselling

While mandatory counselling is intended to support the decision-making of whether to terminate a pregnancy or not, all interviewed counsellors stated that clients are usually already past that process when approaching counselling, and steadfast in their decision to end their pregnancy. In consequence, counselling is largely perceived as an administrative barrier instead of a supportive measure for decision-making.

*“The motivation [of the abortion-seeker] is to get the counselling certificate. For me that is the overwhelming number.”* (Interviewee 01, counsellor, Berlin)

“*Well, I would say that in 95 percent of cases, the women are very decisive. It's no longer a question of whether I can have the child or not. […] They have already given it a lot of thought.*” (Interviewee 08, counsellor, Brandenburg)

“*Most of the women who come in already have one, two or three children. And then it's just a question of family planning being complete or not having the confidence to have another child or simply not wanting to. That's all perfectly legitimate. […] sometimes, depending on your status, financial considerations play a bigger role in saying, okay, now is not the right time.”* (Interviewee 10, counsellor, Brandenburg)

“*So, for many people, we're just a place where you must go to get a piece of paper. […] We're actually just another hurdle. And then you just try to do it as quickly and easily as possible.*” (Interviewee 05, counsellor, Berlin)

It was also pointed out that while in Brandenburg, counsellors can offer video or phone counselling, and the counselling certificate can be sent by mail, in Berlin, generally, the counselling certificate must be collected with the abortion-seeker physically present with documentary proof of identity. This further restricts the provision of the counselling certificate:

*“[…] there are many pregnant people who really have difficulties coming because they are caring for people, working, have a violent partner, have parents who don't know […], a thousand reasons. And Berlin has gone completely backwards. With very few specifically defined exceptions, counselling is mandatory in presence.”* (Interviewee 05, counsellor, Berlin)

However, counselling centres are not always easy to reach in person. Due to information or communication barriers, abortion-seekers frequently arrive at counselling practices without appointments. A counsellor in a small town in Brandenburg explained that they tried to offer consultations right away, being aware that people usually travel long distances:

*“We always try to offer advice immediately, because it's difficult to make an appointment because of the distances involved. Some people don't live in [town with a service]. […] If they are from [other towns], then of course we try to ensure that they don't have to travel too far [for the abortion]. And then, once they are there, we also provide advice.”* (Interviewee 09, counsellor, Brandenburg)

As with most health services, mandatory counselling was also described as subject to restricted public spending. Counselling centres experienced budget cuts, restructuring and shortage of staff and space, sometimes forcing them to move to less central premises.

*“We sometimes must tell women already on a Tuesday: sorry, we don't have any more appointments for this week. And that's [the difficulty] for many, especially those who are on the borderline of whether or not medication is still possible.”* (Interviewee 01, counsellor, Berlin)

*“And we've worked a lot with [organization], but now they're working in the middle of nowhere. So that's why we no longer send the clients there because it really is an hour or more [to get there].”* (Interviewee 07, social worker and client advocate, Berlin)

*“For us, it is actually the case every year that we only get our budget approved in the middle of the year, and until then, we only have advance payments. So, it's not necessarily that unusual that we don't yet know what the situation will be. But the situation [now] is such that there will actually be cuts compared to the previous year's budget, and this is the first time that this has happened.”* (Interviewee 01, counsellor, Berlin)

Another important issue was that, confusingly, not all pregnancy counselling centres issue the required counselling certificate, as is the case with institutions run by the Catholic Church, who are not authorised to provide certificates, in line with the Church’s opposition to abortion. However, those not familiar with the health system may not be aware of these nuances and may attend a counselling session that does not provide the vital paperwork.

*“I once had a woman who came from a church organization and didn't get the certificate. And she asked right at the beginning, “Can I have it?” They just do counselling, but then don't issue the certificate. And she was totally desperate.”* (Interviewee 04, counsellor, Berlin)

### Finding doctors

Our interviews showed that the complex administrative process resulting from criminalisation means that many doctors are uncertain about which services they can or cannot provide. In addition, it is very unlikely that a person can get an abortion from their regular gynaecologist – if they have one – because relatively few gynaecologists provide these services. Their gynaecologists may themselves be prejudiced against abortion, and intentionally not provide a referral.

*“The fact that it's in the penal code means that some people want to protect themselves and say, please bring a referral from perhaps your regular gynaecologist. So, if you haven't been yet, you may have to go there. You may have to communicate, dear gynaecologist, I am unintentionally pregnant, I would like to have an abortion now, please give me a referral slip. That can also be an inhibition threshold.”* (Interviewee 10, counsellor, Brandenburg)

“*But there are still a lot of people who say that my doctor doesn't offer [abortion] and I'm a bit overwhelmed as to where I can go. Sometimes, it probably also has something to do with the attitude or ethical stance of the practices, that they don't think it's a good idea and that they don't give out addresses.”* (Interviewee 04, counsellor, Berlin)

“*It's also kind of annoying for many people who actually have a good relationship with their [regular] gynaecologist and want to have an abortion there, but she doesn't offer it. Because the doctors themselves are still allowed to choose whether to do it at all and which methods to use.”* (Interviewee 10, counsellor, Brandenburg)

This can be even more confusing and intimidating for individuals who face language barriers or lack familiarity with the German healthcare system. One counsellor observed that many people – especially those new to a residential area and searching for a gynaecologist – struggle to find one who offers the necessary services or guidance. This is often due to capacity issues, as many gynaecologists are unable to accept new patients. Even securing an appointment just to confirm a pregnancy can be a significant challenge, as this counsellor explains:

*“I think the more feasible way would be to go to the doctor and discuss the problem with her, so gynaecologists, and not so many have this access. […] They don't use the doctors very much […] because they often can't find a gynaecologist who accepts new patients so quickly and so there is little information flow possible.”* (Interviewee 01, counsellor, Berlin)

*“That´s a good point: whether you have [a regular gynaecologist] at all … ”* (Interviewee 10, counsellor, Brandenburg)

This lack of doctors appears to be partly driven by the retirement of older providers with fewer new doctors entering this field:

*“It's quite tragic because so many people simply can't do [abortion] anymore and so there are fewer and fewer of them. And many are retiring who are still able to do it, there's no one to take their place. […] And that's really tragic, where will that lead?”* (Interviewee 04, counsellor, Berlin).

Because of a shortage of services in Brandenburg, individuals might seek counselling or medical care in Berlin. This creates a predicament for providers in Berlin, as one healthcare worker in Berlin describes:

*“Yes, we are officially not allowed to look after them medically because they are in Brandenburg. We always do it as long as we have the capacity. So, I find it difficult to turn a woman away and we have free places. Yes, if we are full to the brim, then we have to say: I'm sorry, we are not responsible for Brandenburg. Then we give them the addresses for pregnancy counselling or so on in Brandenburg. […] I think that the situation in rural Brandenburg is probably a bit more difficult to find someone, but then you do it in Berlin.”* (Interviewee 03, gynaecologist and counsellor, Berlin)

A medical student and advocate noted that unclear regulations on medical abortion in Germany may lead doctors to offer mostly surgical options. With few providers, this limits patient choice.

*“It depends on whether you do it with medication, whether you do it surgically, whether you want to have anaesthesia, whether you don't want to have anaesthesia, where you are […] You often can't really choose freely because the gynaecologist you see only does surgery. You don't have time because it must happen somehow.”* (Interviewee 12, medical student and advocate, Berlin)

In addition to limiting the choice of procedure, staff at medical practices also may exert discretion in offering appointments. For example, at the doctor’s office, staff at the front desk can play a powerful gatekeeping role:

*“With some women, you say ‘Okay’, but with some women, I can see that [she does not want to]. She says, ‘I want to’, but I say- even if the counselling certificate is three days old- I say, ‘No. You'll come back on Saturday’. […] Of course, 25 years of experience. I see the women who say, ‘I want to’, but her eyes, or, I don't know, just something with her says ‘no’. Then I say, ‘come on, think again’. […] Then I say, ‘you know what? The doctor says we're not starting yet’. Or: ‘I've just seen that we don't have any capacity, operating room capacity, please come back next week’. Okay, she says nothing and leaves.”* (Interviewee 14, gynaecological practice assistant, Berlin)

### Covering the cost of abortion

Interviewees also discussed the complicated cost coverage mechanism if a person is below a certain income threshold and cannot pay for the abortion themself. The federal state will cover the costs in such cases; however, this process is administered by health insurance companies and requires an application to one’s health insurance, if insured, or another agency if not. This is extremely confusing for people unfamiliar with the nuances of the system, and even service providers do not always understand the process, as one social worker reveals:

*“When this law came out, that the health insurance companies … so, actually … so, the health insurance companies … the cost coverage, actually it's not the health insurance companies, it's the state, but still, I don't really understand why the health insurance companies have to do it, why it goes through the health insurance companies.”* (Interviewee 07, social worker and client advocate, Berlin)

Any person seeking abortion cost coverage must request a specific certificate from the health insurance company, usually in person, with documents that prove their income and their residence in the specific state. District health officials noted that personnel at health insurance companies frequently did not help their clients, either because they did not know the procedure themselves, or they were simply not willing or cooperative:

*“The health insurance companies sometimes refuse to cover the costs, which is completely absurd in my eyes, because it's not the insurance companies that pay for it, they actually only do the administration and are paid per case for handling this process, these papers, and have no advantage at all from denying the women this. […] But that always depends very much on the person. The women don't always meet someone who is willing to support them.”* (Interviewee 01, counsellor, Berlin)

Counsellors and client advocates often seek out amenable personnel at health insurance offices and direct their clients to them. This means that access to services is reliant on luck, and on developing personal networks between advocates, providers, and administrators. Counsellors stated that unhelpful health insurance personnel were such a notable stumbling block that they provided abortion-seekers with the legal text that described the process of cost coverage, and a letter from the health department, to persuade the health insurance personnel of their responsibility to administer cost coverage for abortions:

*“We actually also provide the legal text so that we can avoid (the rejection of payment). We have the letter from the Senate, which explains exactly that the health insurance company has nothing to do with the whole story, they don't have to pay any money, it's paid by the state of Berlin, and what is the legal basis, but if they don't even look at it, the [staff] says ‘Goodbye, I don't really feel like doing that’, or has no idea. […] I think it's mostly the case that they are rather unsure.”* (Interviewee 02, counsellor, Berlin)

Interviewees often expressed frustration at this complex cost-coverage process, with one counsellor at a district health office explaining that there was no reason that the district health office itself, which also provides counselling, could not check the financial status of individuals and issue the cost coverage certificate:

*“Maybe that the health insurance companies can be bypassed when it comes to covering the costs, that would be very helpful. So, to be honest, the best thing would be if we could issue this - I'm just going to be cheeky and say that. So, if there would be a wish list for the new year. That would be great, because we are civil servants and I can check that, you can rely on it. And then the costs are covered and that's good. Yes, then I can skip that [insurance step] because I think that's always a bit more difficult.”* (Interviewee 02, counsellor, Berlin)

### Making private and logistic arrangements

In addition to arranging the medical appointments and cost coverage, people seeking abortions must also make the private and logistical arrangements that this process requires. Several providers noted that private organisational issues around children or partners were a concern for their clients.

*“And then the next thing is, if there are already children, how will they be looked after during this time? Especially if they are perhaps still very small. […] the question is whether the existing children are in day care during the day, whether you have the time or whether you must find childcare.”* (Interviewee 10, counsellor, Brandenburg)

*“We don't allow children into our counselling sessions either, so if they come here with their children, they must stay outside. So, they must find someone to look after the children during that time.”* (Interviewee 06, counsellor, Brandenburg)

*“The other thing is always, what kind of support is there from the partner, the ‘sperm donor’? Are they both in agreement about the pregnancy or the abortion? Does the pregnant woman have their support? Or does she perhaps even have to keep it a secret? That can also be difficult. It's difficult to estimate the timing of a medical abortion. On the other hand, in the case of a surgical abortion, she must travel somewhere, perhaps she […] has general anaesthesia. […] Then she needs someone to pick her up after the general anaesthetic. These are all things that can be difficult.”* (Interviewee 10, counsellor, Brandenburg)

It was mentioned that particularly vulnerable groups such as migrants, people with disabilities, addictions, or unhoused people might require additional support services specifically for their needs. Specific organisations catering to these groups may be a first point of disclosure and information, from where they may be guided through the abortion process. Nevertheless, the process may be especially difficult for more vulnerable people. For example, people who use drugs may have problems keeping appointments, making the abortion process extremely difficult, as a social worker observed:

*“And then there are the clients who […] have an addiction. They often say that they want to have an abortion, and because of their illness they are often unable to do so. And then they must attend three appointments. I think […] two appointments with the doctor would be ok, first to see which [pregnancy] week, then again for the medication for the abortion. […] But this whole process, the cost coverage, and then another appointment, and then this and that again.”* (Interviewee 07, social worker and client advocate, Berlin)

## Discussion

This study investigated barriers to abortion in Germany from the perspective of various service providers and client advocates who work at different points on the pathway to abortion. Our findings show that there are multiple individual barriers to abortion access in Germany that are strewn across the entire pathway to care, which, in its complexity, presents a barrier by itself. In our interviews, abortion service providers and client advocates repeatedly mentioned the impenetrability of the German abortion pathway. The described barriers strongly align with results from the CarePreg study among abortion providers in Germany.^22^ Despite the pathway being heavily regulated, accessible information about how to proceed and who offers what services is scant. Many interviewees, including mandatory counsellors, reported that one of their main functions was to provide clients with information about how to navigate the process. This complicated, multi-step pathway is in direct contravention of evidence and best practices on patient-centred care, which promote an integrated approach to abortion services as stated in the WHO (2022) Abortion Care Guidelines.^27^

A pregnancy carried to term takes a considerable toll on the female body and can entail significant consequences for a person´s health, some of them long-lasting or irreversible, and some of them resulting in death.^28^ If prohibitive regulation and legislation force a person to continue an unwanted pregnancy until delivery, they are also forced to experience those harms, which signifies a substantial curtailment of their bodily integrity.^28,29^ Additionally, continuing an unwanted pregnancy can significantly impact the pregnant person’s mental health, especially in the form of antenatal and postpartum depression.^30–32^ The current situation demands that pregnant individuals navigate the complex and overly bureaucratic process, having to coordinate necessary logistic arrangements under extreme time pressure, and to face the continual scrutiny and discomfort of repeatedly disclosing the need for an abortion: in its entirety, this creates an artificial, state-induced barrier to a fundamental reproductive health service with the potential for negative emotional, mental and physical health outcomes. Building on the definition of structural violence as inequitable state actions enacted through often unnoticed legal, political, or economic systems,^25^ our findings suggest that state-imposed barriers to abortion access in Germany should in fact be recognised as a form of structural violence.

A notable example of practices constituting structural violence within the abortion pathway is the mandatory pregnancy conflict counselling. While in no way medically necessary, mandatory counselling has instead been shown to lead to additional appointments, delays in securing a procedure, and distress for the abortion-seeker, and as a result is considered a barrier to abortion care in the existing international literature.^33,34^ This is confirmed by our interviews, where providers and advocates referred constantly to the time, travel, work-leave, and childcare arrangements necessary to attend mandatory counselling. At the same time, it is highly questionable if mandatory counselling fulfils its function: in accordance with our findings, existing research states that pregnant individuals are usually already certain about their decision before they begin the abortion pathway, such that mandatory counselling rarely contributes to decision-making.^35^

Interviewees repeatedly cited concerns about the limited supply of doctors performing abortions in Germany. This is unsurprising, as the health system is overburdened and waiting times for specialists, including gynaecologists, are long, with some doctors not taking new patients.^36–38^ The number of medical facilities reporting abortions in Germany has almost halved from 2,030 in 2003 to around 1,100 in 2021,^39^ and it is roughly estimated that only 1 in 12 gynaecological practices provide abortions.^12^ According to the available data, access to abortion facilities is variable between federal states, and abortion-seekers in states with lower service provision may have to travel to other states.^22,39^ A study analysing requests for telemedicine abortion from Germany showed that 10% of abortion-seekers said they were unable to access abortion through the formal health system because of the distance of the abortion provider and/or lack of transport.^15^ This is also apparent from our data, with medical personnel in Berlin reporting seeing several clients from Brandenburg, where services are fewer, and various providers in Berlin and Brandenburg being concerned about the distance that people had to travel for abortion services.

One reason for the scarcity of doctors providing abortions is a lack of adequate education. In fact, training of doctors in Germany does not include abortion education.^40^ Medical Students for Choice Berlin, a non-profit student organisation addressing the deficit of abortion education in medical training, introduced voluntary workshops in 2015 to fill this gap,^41^ but this means that students who want to learn about abortions must do so on their own initiative. A recent study found that medical students and doctors in Berlin had poor knowledge of both abortion law and medical terminology and procedures.^40^

Additionally, even doctors who perform abortions may not make this information available on their websites, making the search for abortion care even more difficult, based on the research we conducted while locating doctors who provide abortion care. This may be intentional: until July 2022,^42^ it was illegal in Germany to “advertise for abortions”, including doctors stating that they offer abortions on the websites of their practices. Providers who offered information despite the legal prohibition were often harassed, prosecuted and fined.^43^ Whilst the law has changed, the availability of information seems to be slow to improve, likely due to a combination of website maintenance capacity and the remaining stigma surrounding abortion. The reluctance of providers to offer abortions, and the reluctance of those who do to make this information apparent, should be seen in the light of evidence that shows that criminalisation and related stigma negatively impact providers’ willingness to deliver care, especially for highly politicised issues such as abortion.^40,44,45^ Furthermore, because abortion is criminalised, providers and institutions are not obliged to perform abortions due to religious or ethical considerations.^6^ In a Berlin-wide study with doctors and medical students, interviewees widely experienced abortion as a taboo, particularly within the medical discipline, with some interviewees saying that they could not talk openly about abortion with people in their healthcare work environment due to fear of hostility.^40^

Two-thirds of abortions in Germany are performed surgically: in 2021, 52.1% of abortions used vacuum aspiration and 11.4% curettage.^46^ International best practice guidelines insist on the importance of informed choice about the method of abortion and recommend offering medical abortions at least until 12 weeks of gestation, or later.^34,47^ The safety of medical abortion, both in-clinic and by telemedicine, has been well-established;^48,49^ it is a less invasive option for the patient, and a more cost-effective one for the health system.^27,50^ However, in Germany, the legal parameters, administration, and supply of the drugs for medical abortion create uncertainty and additional work for providers, so doctors usually choose not to use them.^51^ The result is a preference for surgical abortions, including curettage, which is no longer considered a safe abortion method.^27^ As our findings highlight, surgical abortion requires an initial consultation with an anaesthetist and the gynaecologist, adding another step before the abortion can be performed. The fact that individuals cannot by default choose a less invasive – and in relation to curettage, even a less risky – procedure represents another example of the structural violence inherent to abortion care affecting physical integrity.

An estimated 60–70% of people seeking abortions make use of cost coverage due to financial hardship.^12^ However, as interviewees at local health offices and client advocates reported, this cost coverage system does not work well, and individuals meeting the criteria for cost coverage have been turned away. In fact, interviewees described that local health offices and the Berlin health department have cooperated to implement informal policies, such as supplying additional documentation, to smooth over dysfunctional processes. Our findings demonstrate that even for people who are eligible for state funding, not including abortion in the covered package of healthcare creates significant additional barriers. This complements existing evidence that the inclusion of abortion care in health insurance coverage is recognised as improving access to care, and its exclusions create inequitable access.^52^

Finally, the multi-step process requires that abortion-seekers constantly arrange and plan for attending appointments and absent themselves from regular responsibilities. Interviewees noted that childcare was one significant issue, as about 60% of people who have abortions in Germany have at least one child.^46^ As abortion is both personally sensitive as well as socially stigmatised, having to continually reveal one’s circumstances to different people in this long process adds further stress and makes the individual vulnerable to discrimination and stigmatisation throughout the pathway. Numerous required appointments could make it especially difficult for abortion-seekers who do not wish to disclose to their families or partners due to stigma, fear or even violence.^53^ In fact, among those in Germany who opted for telemedicine abortions, the need for secrecy (48%) and the wish for privacy (48%) were the most frequent reasons for choosing this route.^15^

As a limitation, our interviews took place only in Berlin and Brandenburg and might not reflect the specificities of abortion barriers within other federal states of Germany. We interviewed only one person who performed the abortion act itself, because we were mostly interested in the multiple steps necessary beforehand; including more interviewees for this last step in the process might have yielded additional information. Further, we included only perspectives of service providers, and additional research focusing on individuals who have experienced abortion would be extremely valuable. However, we believe that the providers´ perspective gives important insights into the structural access barriers to abortion care in Germany, particularly as they have intimate knowledge of a very complex system, and as they mediate between the system and those seeking abortions.

## Conclusion

The pathway to abortion in Germany is arduous, creating administrative and logistical barriers that far outweigh the requirements for the health or safety of the abortion-seeking person. Our findings from abortion providers´ experiences complement the most recent research on access to abortion in Germany that shows that the complex regulatory framework and bureaucracy embedded in abortion access is, in fact, a hindrance to care,^11,15,40,54,55^ as well as the international literature that conceives of restrictive regulatory frameworks as creating structural violence for those who are unintendedly pregnant.^18,54,56,57^ Our findings also support the recommendations by the Commission on Reproductive Self-Determination^58^ to decriminalise abortion and suggest that doing so would significantly reduce access barriers, especially for vulnerable population groups. Key recommendations by the Commission, in alignment with the right to the highest attainable standard of health and the right to equality,^3^ include that abortions in the early stages of pregnancy should be legal, financial coverage should be expanded for low-income individuals, and support for migrants and other vulnerable groups should be enhanced. Crucially, decriminalisation would go a long way towards relieving abortion stigma, enabling better access to information, and abortion care being taught in medical schools, creating a wider social and healthcare environment where providers and clients feel free of shame and fear. Lastly, following the recommendations would align Germany’s abortion access with its international commitments and ensure that the country follows international best practices as outlined by WHO.

## Supplementary Material

Supplementary file S1. Interview Guide - English

Supplementary File S2: Sociodemographic and professional information on interview partners.

Supplementary file S1. Interviewleitfaden - German

## Data Availability

The data are not freely available owing to data protection and consent restrictions, as data contain potentially identifying participant information even after de-identification. We will, however, provide access to the interview transcripts upon reasonable request to the corresponding author.

## Footnotes

* In this paper, we aim to write in a way that is inclusive as possible and thus use gender-neutral language. However, where we quote directly or report findings from other studies, we use the term “women”. Where we speak about our findings and their implications for abortion access, we do so by using the terms “persons/ individuals”, reflecting that people who do not identify as women also seek abortions.
