# Supplementary material for: “She was totally desperate”: understanding the pathway to abortion in Germany through a qualitative study among service providers in Berlin and Brandenburg
Source: Sex Reprod Health Matters. 2025 Jul 23;33(1):2534266. doi: 10.1080/26410397.2025.2534266 (PMC12360047; doi:10.1080/26410397.2025.2534266)
Supplement: Supplementary file S1. Interview Guide - English [file ZRHM_A_2534266_SM8892.docx]

Project MigraH v1.0

Interview Guide

Access to Healthcare for Unwanted Pregnancies

Interview Code: _________

Interview Date: _________

Interviewer: _________

Thank you for taking the time to speak with us today. With your consent, I would like to record this interview.

In our project, we are interested in the experiences of refugees and asylum seekers, i.e., people who have already applied for asylum in Germany or intend to do so. We are investigating how asylum-seeking and refugee women access healthcare when facing unwanted pregnancies. We would like to ask you a few questions about this. Please answer based on your professional experience; we are not asking about your personal experience.

**Sociodemographic Data:**

SD1. How many staff members does your organization have?

SD2. What thematic areas does your organization cover?

- Pregnancy conflict counseling
- Family planning in general (including contraception)
- Domestic violence / family violence
- Specific experiences of sexual violence during flight?

SD3. In which languages do you offer services?

SD4. How many clients does your organization counsel per month (on average)?

SD6. How many asylum seekers and refugees does your organization counsel per month (on average)?

SD5. Position of the interviewee in the organization and main areas of responsibility

SD7. Your age, gender, migration status (yourself or your parents)?

**Questions about Your Experience in Counseling Work:**

1. How do asylum-seeking and refugee women with unwanted pregnancies access your counseling services?

a. How do they learn about your services?

b. What outreach strategies are used?

c. If applicable: who refers them to you?

d. In what week of pregnancy are they (on average)?

2. Do you collaborate with other organizations when counseling asylum-seeking and refugee women with unwanted pregnancies?

a. If yes, why and with whom?

b. If no, why not?

3. What expectations do asylum-seeking and refugee women with unwanted pregnancies bring to your counseling?

4. What topics arise in counseling sessions with asylum-seeking and refugee women with unwanted pregnancies?

a. What role do religious or faith-based beliefs play in the counseling, especially considering that many counseling services are provided by church-affiliated organizations?

5. What challenges do asylum-seeking and refugee women with unwanted pregnancies face when they want to terminate a pregnancy?

a. How do they find a gynecologist to perform the procedure?

b. On average, how long does it take for the procedure to be carried out?

c. How is the procedure billed, or what costs does the person have to bear?

d. How can they access gynecological follow-up care?

e. How are language barriers overcome?

6. How do asylum-seeking and refugee women with unwanted pregnancies perceive the legal framework in Germany (abortion is illegal but not punishable), especially in light of their migration background and often precarious residency status?

7. What are your experiences with post-abortion counseling?

a. Are these services used?

b. What topics are relevant in this context?

8. In your experience, what are the biggest challenges for counselors working with asylum-seeking and refugee women with unwanted pregnancies?

9. How have you adapted your services in recent years to better support the growing number of clients with a migration background?

10. Do you have specific suggestions on how the care for asylum-seeking and refugee women with unwanted pregnancies should be improved?

11. Is there anything else we haven’t asked that you think is important to mention?
