# Supplementary material for: “She was totally desperate”: understanding the pathway to abortion in Germany through a qualitative study among service providers in Berlin and Brandenburg
Source: Sex Reprod Health Matters. 2025 Jul 23;33(1):2534266. doi: 10.1080/26410397.2025.2534266 (PMC12360047; doi:10.1080/26410397.2025.2534266)
Supplement: Supplementary File S2: Sociodemographic and professional information on interview partners. [file ZRHM_A_2534266_SM8891.docx]

| **Interview no.** | **Institution type** | **Location** | **Age**  **decade^[[1]](#footnote-1)^** | **Gender** | **Professional role** | **Highest completed level of Education** | **Work Experience (years)** | **Migrant Background** |
| --- | --- | --- | --- | --- | --- | --- | --- | --- |
| 01 | Counselling  Centre | Berlin | 60 | Female | MD, Counsellor | University | 19 | No |
| 02 | Centre for Sexual Health and Family Planning | Berlin | 60 | Female | Social Worker | University | 41 | No |
| 03 | Public Health department | Berlin | 50 | Female | MD/Counsellor | University | 33 | No |
| 04 | Public Health department | Berlin | 50 | Female | Social Worker/(Counsellor | University | 16 | No |
| 05 | Family planning Centre | Berlin |  | Female | MD, Counsellor | University |  |  |
| 06 | Counselling Centre | Brandenburg | 30 | Female | Social Worker/Counsellor | University | 11 | No |
| 07 | Counselling Centre | Berlin | 30 | Female | Social Worker/Client Advocate | University | 8 | Yes |
| 08 | Counselling Centre | Brandenburg | 50 | Female | Counsellor | University | 24 | No |
| 09 | Counselling Centre | Brandenburg | 50 | Female | Counsellor | University | 25 |  |
| 10 | Counselling Centre | Brandenburg | 20 | Female | Counsellor | University | 3,5 | No |
| 11 | Self Support Group | Berlin |  | Female | Client Advocate |  | 7 | Yes |
| 12 | Activist group | Berlin | 20 | Female | Medical Student/Client Advocate | High School | 3 | No |
| 13 | NGO | Berlin | 30 | Female | Counsellor | University |  | No |
| 14 | Counselling Centre |  | 40 | Female | Medical Assistant | Middle School | 25 | Yes |
| 15 | Counseling Centre | Berlin | 50 | Female | Counsellor | University | 2 | Yes |
| 16 | Language mediation | Berlin | 30 | Female | Client Advocate, Translation coordinator | University | 8 |  |
| 17 | Language mediation | Berlin | 40 | Female | Paramedic, Translator | University | 5 | Yes |

**Supplementary File 2:** Sociodemographic and professional information on interview partners

1. Age of interview partner is presented within the respective decade, to warrant anonymity of the interview partners [↑](#footnote-ref-1)
