# Supplementary material for: “She was totally desperate”: understanding the pathway to abortion in Germany through a qualitative study among service providers in Berlin and Brandenburg
Source: Sex Reprod Health Matters. 2025 Jul 23;33(1):2534266. doi: 10.1080/26410397.2025.2534266 (PMC12360047; doi:10.1080/26410397.2025.2534266)
Supplement: Supplementary file S1. Interviewleitfaden - German [file ZRHM_A_2534266_SM8890.docx]

Projekt MigraH v1.0

# Interview Leitfaden

**Zugang zu Gesundheitsversorgung für ungewollte Schwangerschaften**

Interview Code:

Interview Datum:

Interviewer:

Vielen Dank, dass Sie sich die Zeit für unser heutiges Gespräch nehmen. Mit Ihrem Einverständnis würde ich dieses gerne aufzeichnen.

In unserem Projekt interessieren wir uns für die Erfahrungen von Geflüchteten und Asylsuchenden,

d.h. Menschen, die in Deutschland bereits Asyl beantragt haben, oder vorhaben, dies zu tun. Wir untersuchen, wie asylsuchende und geflüchtete Frauen mit ungewollten Schwangerschaften Zugang zur Gesundheitsversorgung haben. Hierzu möchten wir Ihnen einige Fragen stellen. Bitte beantworten Sie diese aufgrund Ihrer beruflichen Erfahrung – es geht nicht um Ihre persönliche Erfahrung!

# Soziodemografische Daten:

SD1. Wie viele Mitarbeitende hat Ihre Organisation? SD2. Welche Themenbereiche decken Sie ab?

- Schwangerschaftskonfliktberatung
- Familienplanung allgemein (inkl. Verhütung)
- Häusliche Gewalt/ Gewalt in der Familie
- Konkret Sexuelle Gewalterfahrung auf der Flucht?

SD3. Auf welchen Sprachen bieten Sie Services an?

SD4. Wie viele Klient*innen berät Ihre Organisation im Monat (im Durchschnitt)?

SD6. Wie viele Asylsuchende und Geflüchtete berät Ihre Organisation im Monat (im Durchschnitt)? SD5. Position der interviewten Person in Organisation und Hauptaufgabenbereiche

SD7. Ihr Alter, Geschlecht, Migrationsstatus (selbst, Eltern)?

# Fragen zu Ihrer Erfahrung im Rahmen Ihrer Berater*innentätigkeit:

1. Wie erreichen asylsuchende und geflüchtete Frauen mit ungewollten Schwangerschaften Ihr Beratungsangebot?
   1. Woher wissen sie von Ihrem Angebot?
   2. Welche Outreachstrategien werden angeboten?
   3. Ggf: über wen werden sie geschickt?
   4. In welcher Schwangerschaftswoche sind sie (im Durchschnitt)?
2. Arbeiten Sie mit anderen Organisationen zusammen, wenn Sie asylsuchende und geflüchtete Frauen mit ungewollten Schwangerschaften beraten?
   1. Wenn ja, warum und mit welchen?
   2. Wenn nein, warum nicht?
3. Mit welchen Erwartungen kommen asylsuchende und geflüchtete Frauen mit ungewollten Schwangerschaften zu Ihrer Beratung?
4. Welche Themen kommen in Beratungen mit asylsuchenden und geflüchteten Frauen mit ungewollten Schwangerschaften auf?
   1. Welche Rolle spielen religiöse/ Glaubenseinstellungen in der Beratung, vor allem vor dem Hintergrund dass viele Beratungsangebote von kirchlichen Trägern angeboten werden?
5. Vor welchen Herausforderungen stehen asylsuchende und geflüchtete Frauen mit ungewollten Schwangerschaften wenn sie eine Schwangerschaft beenden wollen?
   1. Wie finden Sie eine Gynäkologin, die den Eingriff durchführt?
   2. Wie lange dauert es durchschnittlich, bis der Eingriff vorgenommen wird?
   3. Wie wird dies abgerechnet, bzw. welche Kosten kommen auf die Person zu?
   4. Wie können die Personen gynäkologische Nachuntersuchungen in Anspruch nehmen?
   5. Wie werden sprachliche Barrieren umschifft?
6. Wie erleben asylsuchende und geflüchtete Frauen mit ungewollten Schwangerschaften den rechtlichen Rahmen in Deutschland (Abtreibung illegal aber nicht strafbar), vor allem im Hinblick auf ihren Migrationshintergrund und eigenen prekären Aufenthaltsstatus?
7. Was sind Ihre Erfahrungen mit Beratungen, die nach einer Abtreibung stattfinden?
   1. Werden diese in Anspruch genommen?
   2. Welche Themen spielen hierbei eine Rolle?
8. In Ihrer Erfahrung, welches sind die größten Herausforderungen für Beratende, die mit asylsuchenden und geflüchteten Frauen mit ungewollten Schwangerschaften arbeiten?
9. Wie haben Sie in den letzten Jahren Ihre Angebote angepasst, um der wachsenden Zahl von Klienten mit Migrationshintergrund besser aufzunehmen?
10. Haben Sie konkrete Vorschläge, wie die Versorgung von asylsuchenden und geflüchteten Frauen mit ungewollten Schwangerschaften verändert werden sollte?
11. Gibt es sonst noch etwas, dass wir Sie nicht gefragt haben?
